# Quadriceps Tendon Ruptures: A Clinical Review

**DOI:** 10.7759/cureus.95757

**Published:** 2025-10-30

**Authors:** Ahmad A Quzli, Zeeshan M Ali-Qazalbash, Sondos A Alkhatib, Dileep Karthikappallil

**Affiliations:** 1 Trauma and Orthopaedics, Wirral University Teaching Hospital NHS Foundation Trust, Birkenhead, GBR; 2 Internal Medicine, NHS University Hospitals of Liverpool Group, Liverpool, GBR; 3 Trauma and Orthopaedics, NHS University Hospitals of Liverpool Group, Liverpool, GBR

**Keywords:** diagnosis, eccentric contraction, knee biomechanics, knee extensor mechanism, management, quadriceps tendon repair, quadriceps tendons rupture, rehabilitation, surgical repair

## Abstract

Quadriceps tendon ruptures (QTRs) are uncommon lower limb injuries that lead to pain, loss of active knee extension, and a prolonged rehabilitation course. They typically affect middle-aged or older adults, particularly in patients with comorbidities or degenerative tendon changes, and they most often occur during eccentric contraction when the knee suddenly flexes against an active quadriceps muscle. QTR is a clinical diagnosis. Although history and examination are the cornerstones of diagnosis, imaging is commonly used to confirm the diagnosis and to stage injury severity, with magnetic resonance imaging (MRI) functioning in many centres as the modality of choice to define site, severity, and tissue quality. Management is individualised and depends on injury-related factors such as tear extent, chronicity, tissue quality, and patient-related factors such as age, comorbidities, and functional demands. Selected partial-thickness tears in low-demand patients may be treated non-operatively, whereas operative repair is generally the mainstay for complete ruptures, high-grade partial tears with functional deficit, and chronic presentations. Evidence indicates broadly similar outcomes with transosseous tunnel and suture anchor repair. Current research into biological allograft augmentation and minimally invasive approaches is ongoing and may influence future practice.

## Introduction and background

Quadriceps tendon rupture (QTR) is relatively rare, with an incidence of approximately 1.37 per 100,000 [1-3], yet it can result in substantial disability because active knee extension is compromised, and the quadriceps is central to mobility. Early recognition and appropriate management are therefore essential to preserve function and to avoid chronic impairment. QTR most often arises in middle-aged to older adults with comorbidities or degenerative tendon changes, and in younger patients after direct trauma [4]. A frequent mechanism is eccentric contraction, whereby the quadriceps contracts while lengthening and the knee suddenly flexes against an active muscle, creating high tensile load across the tendon. Because the condition is uncommon, limited awareness can contribute to missed or delayed diagnosis, which consequently results in inadequate or inappropriate patient management.

This narrative review aims to summarise contemporary evidence on QTR to support day-to-day clinical decision-making. It describes relevant anatomy, biomechanics, and recognised risk factors. It outlines the typical clinical presentation and the role of imaging in diagnosis and staging. It summarises current management strategies, including non-operative treatment, primary repair, augmentation, and reconstruction of chronic cases. It highlights key principles of postoperative rehabilitation and the expected prognosis. It also discusses complex scenarios such as chronic rupture, bilateral rupture, and rupture after total knee arthroplasty, and it reviews recent advances and areas for future research.

Methods

This narrative review draws on iterative searches of MEDLINE (Medical Literature Analysis and Retrieval System Online) and PubMed with manual screening of reference lists from key clinical and biomechanical papers. Searches were updated through September 2025, and no formal date restriction was imposed. Studies were selected for their direct clinical relevance to QTR and their influence on current practice, rather than through a predefined systematic protocol with rigid inclusion and exclusion criteria. The review focuses on evidence that informs practical decision-making in QTR. This includes mechanism and risk factors, bedside recognition, the role of ultrasound and magnetic resonance imaging (NRI) in diagnosis and staging, indications for non-operative management, indications for operative repair, comparison of transosseous tunnel and suture anchor fixation, augmentation and reconstruction in chronic rupture, rupture after total knee arthroplasty, rehabilitation, and prognosis. Preference was given to peer-reviewed cohort studies, multicentre series, and systematic reviews that reported functional outcomes or complication rates, together with biomechanical and technical work that informs surgical decision-making. Single-patient case reports were included only when they addressed rare but clinically important situations. No formal risk-of-bias assessment was undertaken, and no meta-analysis was performed, which is consistent with the narrative scope.

## Review

Anatomy and biomechanics

**Figure 1:**
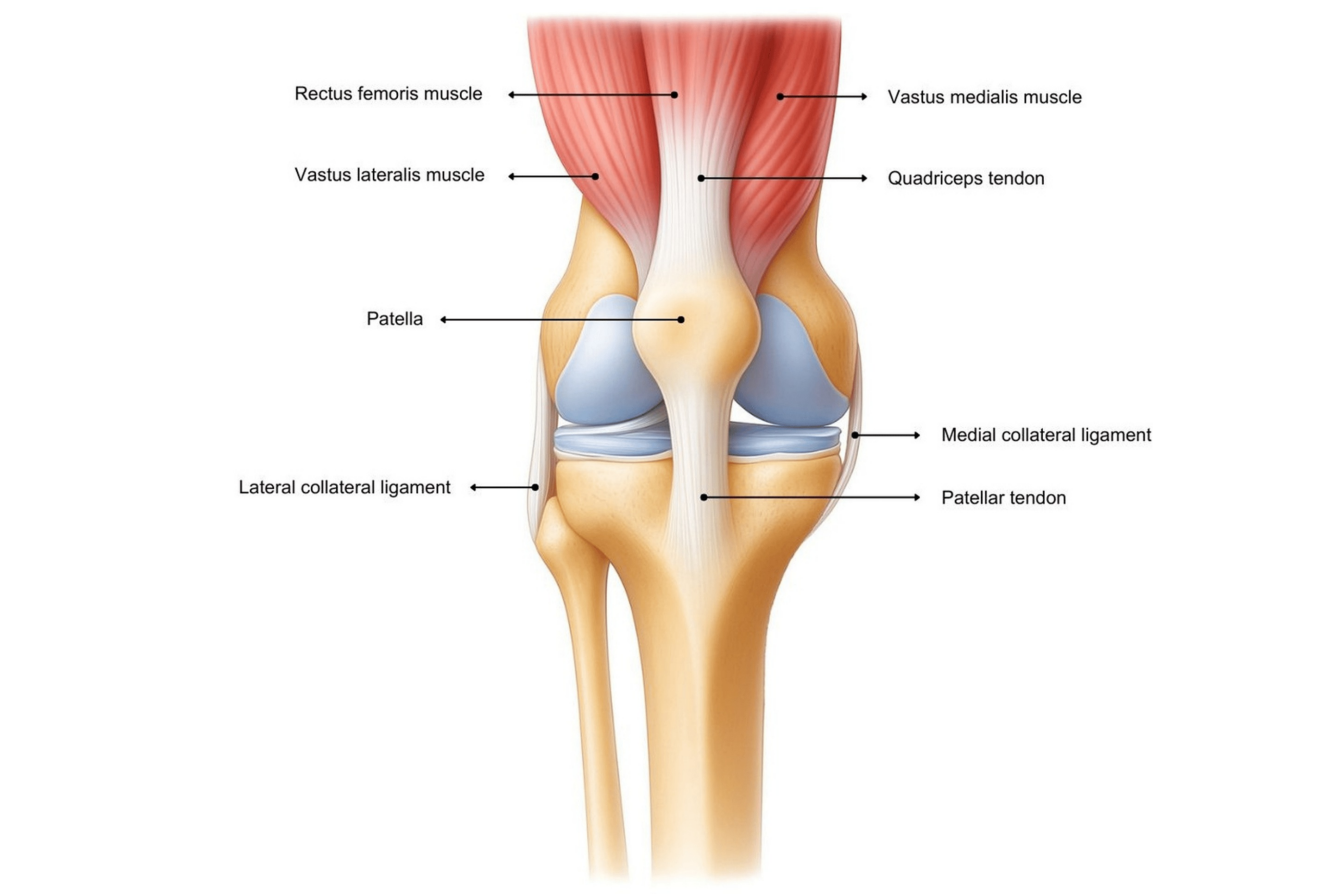
Anterior view of the knee and quadriceps tendon Image Credit: Authors

**Figure 2:**
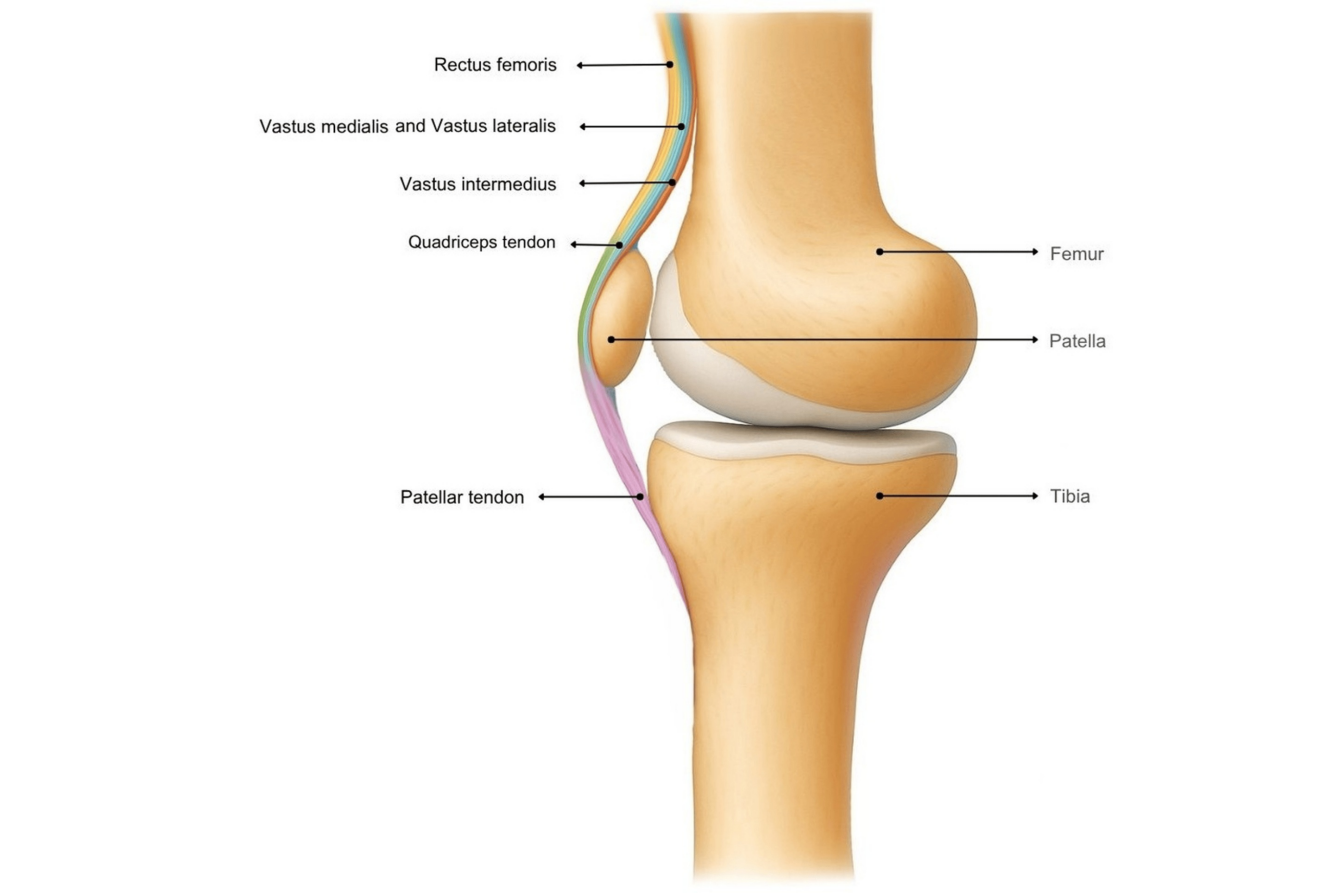
Layers of quadriceps tendon Image Credit: Authors

The quadriceps tendon is integral to the knee extensor mechanism. It transmits force from the quadriceps to the patella and then to the tibial tuberosity via the patellar tendon, producing knee extension required for walking, running, standing from a seated position, and ascending stairs [3]. The tendon forms from the confluence of rectus femoris, vastus medialis, vastus intermedius, and vastus lateralis, which coalesce into a common tendon that inserts into the superior pole of the patella, as shown in Figure 1 [3]. Histologically, the tendon comprises dense fibrous connective tissue with collagen fibres aligned to resist tensile load, and a trilaminar arrangement is classically described with a superficial layer from rectus femoris, an intermediate layer from vastus medialis and vastus lateralis, and a deep layer from vastus intermedius, as shown in Figure 2 [5]. Vascular supply arises from branches of the femoral system; a relatively hypovascular region near the osseotendinous junction may predispose to degeneration in older patients and in those with medical comorbidities [1]. This multilayered architecture and the relatively hypovascular enthesis help explain the predilection for failure at the patellar insertion and support fixation strategies that restore the native footprint.

Aetiology and risk factors

QTR commonly follows abrupt eccentric loading against a flexed knee during activities such as jumping, running, or stumbling [4]. Men over 40 years are most frequently affected, with consistent associations to systemic conditions that promote tendon degeneration. Degenerative change, microtears, and chronic overuse can reduce tensile integrity over time [2]. In younger, otherwise healthy individuals, a discrete traumatic mechanism is typical, often a fall onto a flexed knee or a direct blow to the anterior thigh [4]. Prior anterior knee pain may indicate underlying tendinopathy. Approximately 50% of the tendon’s fibres must be disrupted before a rupture can occur [2].

Systemic factors associated with QTR include diabetes mellitus, as it affects the vascularity of the tendon, reducing its ability to heal, and chronic kidney disease, which contributes to metabolic disturbances that weaken the collagen structure of the tendon, particularly in patients undergoing dialysis [4]. Obesity and metabolic syndrome augment tensile demand and low-grade inflammation, and hyperparathyroidism alters calcium metabolism; together, these conditions are recognised modifiers of rupture risk through their effects on tendon quality [4].

**Figure 3:**
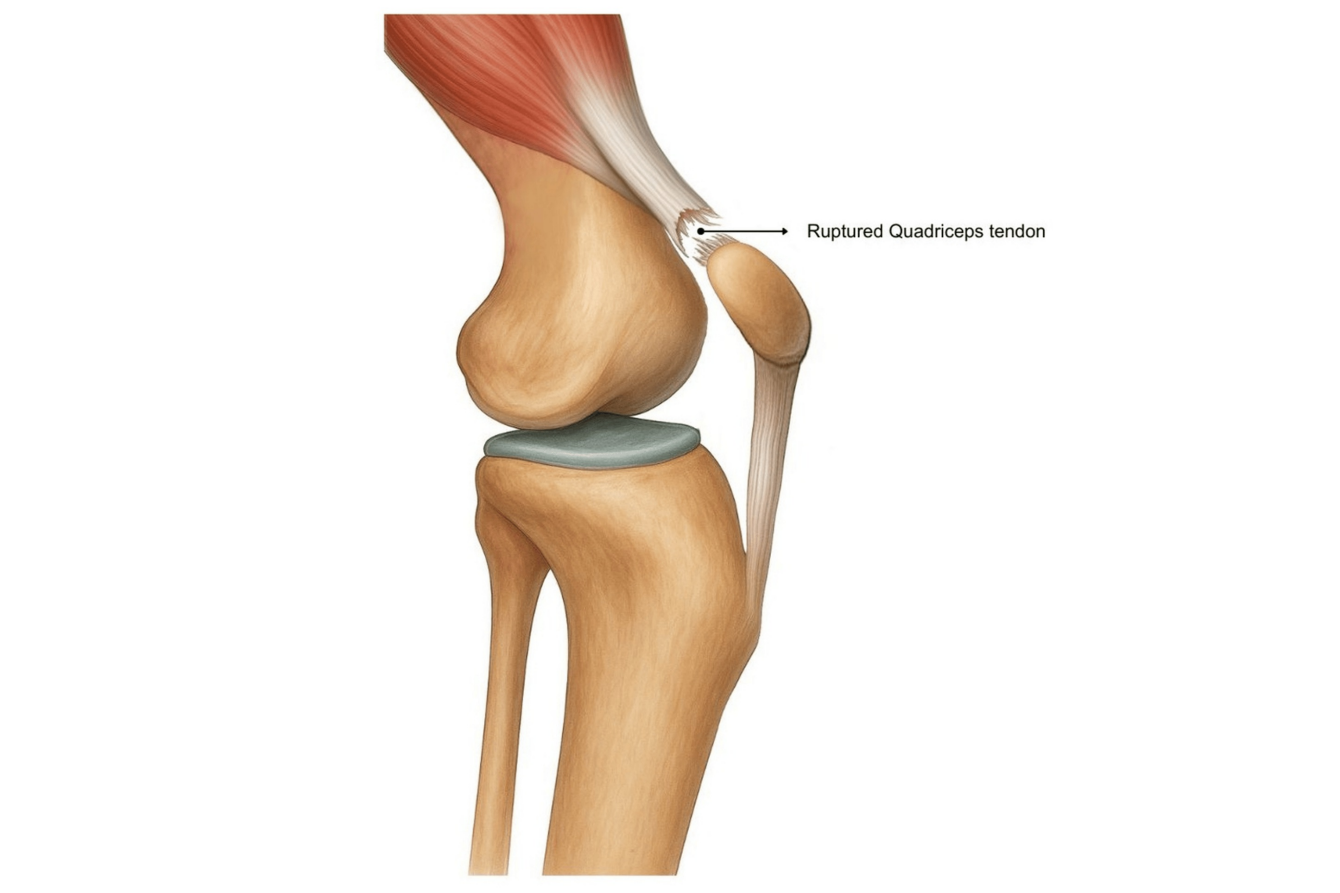
Quadriceps tendon rupture Image Credit: Authors

Consistent degenerative pathology is reported across the literature, including classic histology and rare bilateral presentations [6-8]. Exposure to corticosteroids and fluoroquinolone antibiotics has been linked with tendon weakening and spontaneous rupture, particularly with combined exposure [9,10]. Peritendinous or intra-articular corticosteroid injections have also been associated with increased rupture risk in susceptible tendons, with reported rupture rates of 20-33%, and should be used judiciously [11]. QTR is a recognised complication after knee surgery, such as total knee arthroplasty or anterior cruciate ligament reconstruction, as altered biomechanics, surgical trauma, scar tissue formation, and post-operative immobilisation can reduce tendon strength and cause rupture [12,13]. A conceptual rupture schematic is shown in Figure 3.

Clinical presentation

Patients with QTR typically report a sudden, painful event, often a stumble or attempted recovery from a fall, sometimes with a popping or tearing sensation. Symptoms include anterior knee pain, swelling, bruising around the superior patella or anterior thigh, difficulty weight bearing, and loss of active knee extension [11]. Examination may reveal a palpable suprapatellar defect, although acute swelling can obscure this sign. Some straight-leg raise may persist via retinacular continuity, but active extension is reduced or absent [2,4]. The patella may be low-lying, termed patella baja, due to proximal tendon rupture with retraction. Inability to maintain passive extension against gravity may also be present [11]. Initial misdiagnosis remains reported, particularly in partial or degenerative tears, as highlighted in contemporary reviews [2,4].

Diagnosis

QTR is primarily a clinical diagnosis supported by targeted imaging. In chronic presentations, imaging also informs operative planning by indicating when augmentation or reconstruction is likely if tissue quality is poor [14]. Plain radiographs in anteroposterior and lateral views may demonstrate soft tissue swelling, a superior pole avulsion fragment, and patella baja; these films are useful to identify associated bony injury, but the absence of such findings does not exclude QTR. Ultrasound is widely accessible and can rapidly distinguish partial from complete tears at the bedside, although it is operator dependent and its accuracy varies between settings [15-18]. MRI is the modality of choice in many centres to confirm the site and grade of rupture, to assess retraction and tissue quality, and to facilitate pre-operative planning; it can also demonstrate associated injury to surrounding structures that influence surgical decision-making [4,16,17].

Classification

QTRs are classified by extent, chronicity, location and imaging grade as outlined in Table 1, and this framework guides care by indicating when surgery is required, whether primary repair is feasible or augmentation is likely, how fixation or reconstruction should be planned, and what degree of retraction and tissue quality to anticipate on MRI or ultrasound, with key imaging findings summarised in Table 2 [2,4].

**Table 1:** Clinical and radiological classification of quadriceps tendon ruptures (practical scheme) Content synthesised from: [2,4,16,17]

Category	Classification
Extent of rupture	- Partial
	- Complete
Chronicity	- Acute (<3 weeks)
	- Subacute (3 to 6 weeks)
	- Chronic (>6 weeks)
Location	- Osseotendinous junction at patellar insertion (most common)
	- Tendon body mid-substance tears (often degenerative)
	- Myotendinous junction (less common)
Radiological	- Grade I: tendinopathy or mild strain with no fibre disruption
	- Grade II: partial-thickness tear
	- Grade III: full-thickness tear with retraction, haematoma or fluid at the rupture site; patella baja may be present

**Table 2:** MRI and ultrasound features by grade of quadriceps tendon injury Content synthesised from: [15,17,18]

Grade	MRI Findings	Ultrasound Findings
I	Thickened tendon with signal change consistent with tendinopathy and no fibre disruption.	Mild hypoechoic change with preserved tendon continuity.
II	Focal discontinuity of some tendon fibres with surrounding oedema, consistent with a partial-thickness tear.	Focal hypoechoic defect within the tendon with partial preservation of continuity, consistent with a partial-thickness tear.
III	Complete disruption of all tendon layers with retraction and haematoma or fluid at the rupture site, and patella baja may be seen in the appropriate clinical context.	Full-thickness hypoechoic gap with tendon retraction and loss of continuity during quadriceps contraction.

Management

Management is individualised according to injury-related factors such as tear extent, chronicity, and tissue quality, and patient-related factors such as age, comorbidities, and functional goals [2-4,11].

Non-operative treatment is limited to carefully chosen patients. It is most appropriate for partial tears where the extensor mechanism is preserved, in patients with only mild symptoms, or in those whose comorbidities or low physical demands make surgery less suitable [19]. Standard care involves immobilisation of the knee in full extension, either with a cast or brace, for about four to six weeks, followed by progressive rehabilitation. Physiotherapy begins with a gentle range of motion exercises, with strengthening of the quadriceps introduced gradually as mobility improves [11]. Outcomes can be satisfactory in low-demand patients, although younger or more active individuals often achieve better function with operative repair [2,20].

Operative repair is generally the mainstay of treatment for complete ruptures, high-grade partial tears with functional deficit, failed conservative care, and chronic ruptures. Better outcomes are seen with early surgery, ideally within the first three weeks, as delayed repair is associated with tendon retraction and poorer tissue quality [2,4,6]. Several techniques are described in the literature. In transosseous repair, sutures are passed through the tendon and tied through bone tunnels in the patella. In suture anchor repair, the tendon is fixed to the superior pole of the patella using anchors. Comparative studies show that both methods provide similar strength, but anchor repair may shorten operative time and reduce bone loss [21,22]. A recent comparative systematic review reports no clinically meaningful difference, including re-rupture, between anchor and transosseous repair [23]. The main practical differences between these fixation strategies are outlined in Table 3.

**Table 3:** Practical comparison of transosseous tunnel repair and suture anchor repair in primary quadriceps tendon rupture Content synthesised from: [21-23]

Aspect	Transosseous tunnel repair	Suture anchor repair
Fixation concept	High-strength sutures are passed through the tendon and tied through longitudinal bone tunnels in the patella to restore the footprint.	Suture anchors are inserted into the superior pole of the patella and their sutures are passed through the tendon stump to secure it back to bone.
Bone work	Requires drilling full-thickness patellar tunnels.	Requires anchor sockets and generally removes less patellar bone overall.
Operative time	Can be longer because of tunnel drilling and suture passage.	May shorten operative time by streamlining fixation.
Clinical outcomes and re-rupture	Restores active extension and function with low reported re-rupture rates.	Patient-reported outcomes and re-rupture rates are broadly comparable, with no consistent clinically meaningful difference.
Practical considerations	Implant-free, familiar and cost-effective, but relies on patellar tunnels.	Useful where bone quality is limited or where large tunnels are less desirable, but requires anchors.

When tendon tissue quality is poor or a gap persists, augmentation can improve construct stability. Options include reinforcement with cerclage wires, autograft such as semitendinosus tendon, or synthetic mesh, which can improve stability and reduce the risk of re-rupture [2,4].

Chronic ruptures that present more than three weeks after the injury are challenging to manage because of scarring, shortening, and degeneration; direct repair may not be possible, requiring reconstruction. Reconstructive options include V-Y lengthening, autograft or allograft reconstruction such as hamstring or Achilles tendon, dermal allograft augmentation, or synthetic mesh [14,16]. In patients who rupture their quadriceps tendon after total knee arthroplasty, reconstruction of the tendon is required to restore the active knee extensor mechanism, and these patients are at high risk of re-rupture and long-term extensor weakness [12-14]. Although outcomes have improved with modern reconstructive options, results are still less predictable in these patients [16].

Postoperative care

The early goals after repair are to protect the construct, prevent stiffness, and restore quadriceps activation. Rehabilitation is usually delivered in phases, but progression is individualised according to intra-operative findings, tissue quality, and fixation strength [4]. In the early phase, which is typically the first zero to six weeks after surgery, the knee is immobilised in extension in a brace, usually full-time for several weeks. Weight bearing is generally allowed as tolerated with the brace locked in extension. A controlled increase in passive and then active-assisted knee flexion often begins after the first two to three weeks, provided the repair is judged secure. The aims in this period are protection of the repair, control of pain and swelling, and prevention of early stiffness around the patella [4]. In the intermediate phase, roughly 6-12 weeks after surgery, the brace is gradually unlocked and the patient progresses towards unassisted ambulation. Active range of motion exercises are advanced towards functional flexion. Closed-chain and then open-chain quadriceps strengthening are introduced as tolerated. The aims in this period are recovery of functional range, normalisation of gait, and establishment of early strength [4]. In the later phase, beyond three months, higher-level strengthening, proprioceptive work, and task-specific drills are added. Most patients regain day-to-day function by four to six months, although return to heavier activity, sport, or occupational loading may extend to 6-10 months in higher-demand cases [24-26]. The rate of progression depends on repair robustness, tendon quality, comorbidities, and patient goals [24-26]. A phased, goals-based rehabilitation plan that is tailored to the individual repair is preferable to a single fixed timeline [4,24-26].

Outcomes and prognosis

Non-operative care can succeed in carefully selected partial tears, although higher-demand patients may have residual weakness [19]. Early repair of complete tears generally restores active extension and range of motion with high rates of return to baseline activities [24,25]. Contemporary series report low re-rupture rates of about 2-6% and generally favourable outcomes, with most patients resuming daily activities by four to six months and sports by 6-10 months when appropriate surgical technique and post-operative rehabilitation have been undertaken [25,26]. Postoperative complications include re-rupture, thromboembolic events, anterior knee numbness, stiffness, and extensor lag, where greater than 5 degrees has been reported in about 11% in some series [25,27]. In delayed or chronic cases, such as those presenting more than six weeks after injury or with poor tendon quality, the prognosis is less predictable. They might require complex reconstruction with tendon grafts or mesh with a high chance of complications such as extensor lag, persistent weakness, or stiffness [28]. Delayed diagnosis, poor tissue quality, comorbidities such as diabetes or chronic kidney disease, non-compliance, and inadequate postoperative rehabilitation are poor prognostic factors [25,27-30]. Recent multicentre cohort data also show no difference in clinical failure between anchor and transosseous repairs, which is consistent with the previous literature [31].

Special considerations

QTRs can be particularly challenging in certain situations, and specific approaches are required for diagnosis, management, and rehabilitation. Bilateral QTRs, although extremely rare, are often associated with systemic conditions such as chronic kidney disease, hyperparathyroidism, and chronic steroid use [29,30]. Because the symptoms occur bilaterally, the diagnosis can be missed or delayed; therefore, a high level of clinical suspicion supported by imaging is essential to identify bilateral cases. Operative management is usually indicated and may require additional procedures depending on the patient’s fitness and level of activity. Chronic ruptures, defined as those with more than six weeks’ delay in diagnosis, are complicated by fibrosis, tendon retraction, and poor tissue quality, which make direct repair more difficult. Reconstructive options such as V-Y advancement, autografts, allografts, or synthetic mesh may be required, though the outcomes are less predictable and are associated with a higher risk of extensor lag and chronic weakness [28]. QTRs that occur after total knee arthroplasty present management challenges due to altered knee anatomy, the presence of scar tissue, and poor native tissue quality. These are generally managed with reconstruction of the extensor mechanism rather than direct repair, but the prognosis is unpredictable and the risk of complications, including stiffness and chronic extensor dysfunction, remains high [32,33].

Recent advances and future directions

Recent developments in surgical management, such as the use of suture anchors and minimally invasive techniques, have provided stronger repairs and the potential for faster recovery [21]. Advances in imaging, particularly high-resolution MRI, have also improved early diagnosis and pre-operative planning, aiding in surgical decision-making and treatment optimisation [17,18]. As structured rehabilitation is essential for optimal recovery, wearable technology and application-based rehabilitation tools are increasingly being incorporated to provide individualised monitoring and care [34].

Beyond conventional repair techniques, biologic augmentation represents a promising frontier in the management of QTR. Laboratory and preclinical studies suggest that platelet-rich plasma (PRP) can enhance tendon healing by stimulating angiogenesis, modulating inflammation, and promoting collagen synthesis [35]. Stem cell-based therapies, particularly tendon-derived stem/progenitor cells and mesenchymal stem cells, have also shown potential in regenerating tendon tissue and improving biomechanical strength in experimental models [36]. While these approaches remain largely investigational and lack large-scale clinical validation in quadriceps tendon injuries, they highlight an important direction for future research aimed at improving tendon healing, reducing re-rupture risk, and accelerating functional recovery.

Looking ahead, future research is directed toward optimising repair techniques, refining biologic therapies, and developing early mobilisation protocols that may further improve functional outcomes [22,25].

Limitations

This article is a narrative review rather than a formal systematic review. Although targeted database searches and manual reference screening were performed to identify relevant studies, study selection and data extraction were performed by the authors without independent duplicate screening; no formal risk-of-bias assessment was undertaken, and no meta-analysis was performed. Much of the available literature consists of retrospective case series with small samples, heterogeneous outcome measures, and short follow-up, and prospective data remain limited. The findings should therefore be interpreted with appropriate caution.

## Conclusions

Although QTRs are rare, they are severe injuries that can lead to significant dysfunction; hence, early diagnosis and management are essential to ensure satisfactory outcomes. QTR is primarily a clinical diagnosis, supported by imaging to avoid missed or delayed diagnosis. Patient-related factors and injury-related factors should be taken into consideration to guide treatment options and decisions to ensure individualised care. However, operative management remains the cornerstone for complete ruptures. Advances in surgical techniques and rehabilitation have improved outcomes, but chronic ruptures and comorbidities continue to present major challenges. Emerging research into biologic augmentation and minimally invasive approaches offers promising avenues for enhancing tendon healing and long-term function.
